# Leaching and VOC Emission Tests of Polymer Composites Produced from Post-Consumer Waste in Terms of Application in the Construction Sector

**DOI:** 10.3390/ma14133518

**Published:** 2021-06-24

**Authors:** Mateusz Kozicki, Katarzyna Guzik, Halina Deptuła, Justyna Tomaszewska

**Affiliations:** Building Research Institute, Department of Thermal Physics, Acoustics and Environment, 00-611 Warsaw, Poland; k.guzik@itb.pl (K.G.); h.deptula@itb.pl (H.D.); j.tomaszewska@itb.pl (J.T.)

**Keywords:** recycling, composite, polypropylene, polyethylene, cellulose fibres, leaching, VOC emissions, construction products

## Abstract

One of the existing priorities of the European Union is to search for rational waste management and to keep such waste in the economic cycle, while meeting the highest safety requirements. The paper presents the results of environmental tests of composites based on the polyethylene (rPE) and polypropylene (rPP) matrix and reinforced with cellulose fibres (newsprint, NP). Raw materials were obtained by recycling post-consumer waste such as beverage bottles and newsprint. The composites were tested for their potential use as materials in cladding panels and acoustic barriers. Given that normative documents for these products do not define specific environmental requirements, the composites were tested for the release of dangerous substances, such as anions of inorganic compounds, heavy metals, volatile organic compounds (VOCs), and their impact on the environment. A detailed in-depth analysis of the mechanisms of release of substances (diffusion, dissolution, surface leaching and depletion) from the rPP/NP composite into surface water, groundwater and soil was carried out. In turn, emission of VOCs from the rPE (low-density:high-density (LD:HD)—50:50) and rPE (LD:HD—30:70) composites into indoor air was also carried out. Raw materials in the form of granulates and loose cellulose fibres, used to produce the composites, were also tested for their environmental impact.

## 1. Introduction

The mitigation of negative effects resulting from the overexploitation of natural resources is currently a global challenge. One of the ways of mitigating these effects is to close the circulation of raw materials in the economy, which is a measure implemented by the European Union Member States as part of the economic transition towards a circular economy (CE) [1,2,3], and legislative changes introduced as part of a new European order called the Green Deal [4,5]. The main idea behind the CE concept is the existence of an industrial economy that is by definition renewable. Its primary goal is to ensure the efficient circulation of raw materials, energy, labour and information so that the human and environmental resources involved can be restored [6,7,8,9].

Plastic waste is a valuable resource that can be used as a raw material for new products or energy recovery when recycling is impossible. One of the biggest challenges in terms of the recycling of plastics is to obtain a high-quality recyclate compared to the primary raw material, whilst ensuring its low price [10,11]. Primary consumers from the plastic processing industry, construction material industry, and other industries will be interested in using recycled plastics provided that they are produced according to well-defined specifications that ensure the uniformity and repeatability of their properties [12]. The development of certification systems for recycled plastics aims to increase the confidence of the industry and consumers in this group of materials, and be an important tool in this regard [13]. This proposal is included in the European Strategy for Plastics in a Circular Economy [14].

Polyethylene (PE) and polypropylene (PP), make up about half of all plastics processed in the EU [15]. Increasing effort is being put into investigations of different plastic waste fractions, their composition, and the associated implications for recycling [16,17,18]. Elucidating fundamental structure–property relationships that govern their technical utility are key for their more widespread use. There is a considerable number of publications focused on the development of composites based on recycled PP and PE matrices and recovered fibres extracted from various species of trees, grass, and crops as reinforcement [19,20,21].

*New circular economy through the valorisation of post-consumer plastic waste and reclaimed pulp fibre* is the title of a research project, known by its acronym Ceplafib [22,23] and financed by the European Commission under the Life programme. The aim of the project is to develop technologies for processing post-consumer waste in the form of beverage bottles and newsprint in order to obtain cellulose fibre-reinforced polymer composites with competitive properties in relation to conventional materials.

In relation to the recycled plastic matrices, a specially developed in-house technology allows us to produce different polymer matrices, i.e., PP, high-density polyethylene (HDPE), low-density polyethylene (LDPE), and its mixtures (PE HD/LD), all in a consistently high quality obtained 100% from post-consumer waste streams. When those two components were merged in appropriate proportions and by the addition of special binders (i.e., coupling agents) and impact strength improvers, completely new materials, adapted to the final needs of the application or product, were created as part of the Ceplafib project.

The paper presents the results of environmental tests of composites based on the polyethylene (rPE) and polypropylene (rPP) matrix, reinforced with cellulose fibres (newsprint, NP), which were developed under the Ceplafib project. The aim of the project is to develop technologies of processing post-consumer waste (pellets/raw materials) in the form of beverage bottles and newsprint in order to obtain cellulose fibre-reinforced polymer composites with competitive properties in relation to conventional materials. OMAPLAST (a Slovenian company responsible for urban plastic waste recycling) has, over the course of long-standing R&D, created a well-established recycling process for solid plastic waste recovery. The Finnish company ECOPULP was responsible for urban paper with short cellulose fibers (newsprint) waste recycling. The characteristics of the rPE/NP and rPP/NP composites, including mechanical, thermal and morphological properties were published by Fajs P. et al. [24] and Bogataj V.Z. et al. [25].

The composites were tested for their potential use as materials in cladding panels and acoustic barriers. The methods used to describe plastic recyclates are specified in PN-EN 15344:2010 [26] and PN-EN 15345:2010 [27]. These standards describe the required properties of recyclates and the methods for determining them. They do not, however, define environmental requirements for these types of material.

The release of dangerous substances from construction products in contact with water during use poses a potential hazard to the environment (soil, surface water, groundwater). Knowledge of the characteristics of leaching processes makes it possible to predict and evaluate the release of substances during the intended use of the product. The specification CEN/TS 16637-2:2014 Construction products—Assessment of release of dangerous substances—Part 2: Horizontal dynamic surface leaching test [28] describes the methods for determining the leaching of dangerous substances from construction products, including:

- the dynamic surface leaching test (DSLT) used to determine the release per unit surface area as a function of time of inorganic and/or non-volatile organic substances from a monolithic, plate- or sheet-like product, when it is put into contact with an aqueous solution (leachant). This test is a parameter-specific test focusing on identifying and specifying parameter specific properties tested under specified conditions. It is not aimed at simulating real situations;
- the GLHC (Method for Granular construction products with Low Hydraulic Conductivity) test is a variation of the DLST test for porous construction products with low hydraulic conductivity.

The GLHC test results provide the basis for determining the mechanism of release of substances from the tested materials following the analysis of a number of indicators calculated based on the results of testing the concentration of substances in eluates obtained for individual test steps (steps 1–8). These indicators are presented in the Discussion section. The prediction of long-term leaching of dangerous substances is important in terms of the evaluation of the behaviour of a construction product in its entire life cycle.

In order to evaluate this process by extrapolation, knowledge of the release mechanisms is required. The release mechanism is a physical and chemical process that describes the release of dangerous substances into a leachant.

Generally, two main types of release mechanism can be distinguished: diffusion and dissolution. The release mechanism can be determined based on the DSLT test results presented on a time vs. standard concentration graph. The diffusion-controlled release mechanism is characterised by linear dependence on time. The leachant exchange frequency adopted in the aforementioned specification is ensured by a three-step process. The concentration in the second and third steps is twice the concentration in the preceding step. For the dissolution-controlled release mechanism, concentration is constant and time-independent.

In practice, the mechanism of release from construction products is a combination of diffusion and dissolution. If solubility is controlled by the pH value, it is disturbed by pH changes during the test. As a result, in practice, the dissolution-controlled release mechanism may be less unambiguous than diffusion. The analysis of the DSLT test results, however, focuses on whether the release mechanism is diffusion-controlled. If no diffusion is identified, the presence of dissolution is tested. If dissolution is pH-dependent, it may occur for limited pH changes. If pH changes are significant, dissolution cannot be considered, and it is assumed that release is controlled by an unidentified mechanism (it may include pH-dependent dissolution or the mixed diffusion–dissolution process).

If diffusion is found to be the main release mechanism, surface leaching and/or depletion may be identified as possible secondary mechanisms affecting the long-term process. In established conditions (limited pH changes), surface leaching may also be found in combination with unidentified leaching processes.

Tests for the release of dangerous substances from construction materials used indoors are based on PN-EN 16516+A1:2020-12 [29]. The tests refer to emissions of volatile organic compounds and volatile aldehydes from construction products into indoor air. The results obtained are related to rules and regulations in the country of application. There are several European Union countries that do not have internal regulations governing the emissions of dangerous substances from construction materials. Some countries use voluntary building rating systems, such as Blue Angel^®^ (Bonn, Germany), EMICODE^®^ (Düsseldorf, Germany) and M1^®^ (Helsinki, Finland), while the German Committee for Health-Related Evaluation of Building Products (AgBB) and the French volatile organic compound (VOC) regulations with their VOC emission classes (A+ to C) are mandatory rating systems in these countries [30]. For several years now, working parties and committees of the European Commission have been involved in the systematisation of this field in all Member States.

## 2. Materials and Methods

For environmental tests, secondary raw materials obtained from the recycling of beverage bottles—rPE and rPP—newsprint (NP) and the composites produced from them were used; see Table 1. The paper [25] presents a method for producing the secondary raw materials and their properties. The thermoforming process developed under the Ceplafib project [22] was used to obtain polypropylene matrix composites reinforced with cellulose fibres (rPP/NP) and polyethylene-based composites reinforced with cellulose fibres (rPE (HD:LD 50:50) + NP, rPE (HD:LD 70:30) + NP).

### 2.1. Testing for the Release of Dangerous Substances into the Environment

#### 2.1.1. Recycling Materials

**Figure 1:**
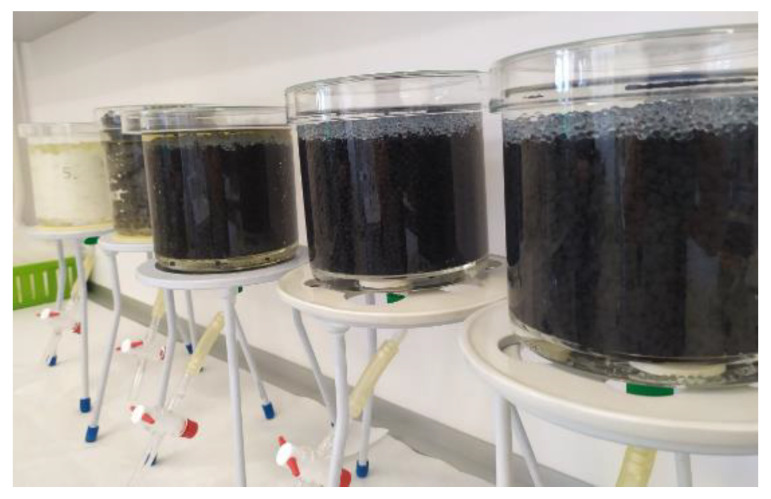
Testing for the release of ions and heavy metals from porous materials during leaching.

For porous materials (rPE, rPP and NP), substances were released as part of the leaching process. The products were pressed lightly and placed in cylindrical vessels. Exposure to the leachant occurred only for the upper layer—see Figure 1. Tests for the release of ions and heavy metals were performed for polymer materials (rPE and rPP) and cellulose (NP) in order to evaluate their environmental impact in terms of their reuse in composites deployed in the construction sector.

In order to evaluate the environmental impact of recycling materials, tests for the release of ions and heavy metals were performed as described in CEN/TS 16637-2:2014 [28]. The purpose of the tests was to determine the characteristics of the leaching process and to evaluate the release of environmentally harmful substances. The tests involve the exchange of leachant at the intervals given in Table 2.

The following physical properties were measured in the collected eluates:

- pH acc. to PN-EN ISO 10523:2012 [31];
- electrical conductivity acc. to PN-EN 27888:1999 [32];
- Inorganic constituents were also determined, i.e.,
- total organic carbon (TOC) acc. to PN-EN 1484:1999 [33];
- anions (fluorides, chlorides, bromides and sulphates) acc. to PN-EN ISO 10304-1:2009 [34];
- heavy metals acc. to PN-EN ISO 11885:2009 [35];
- mercury acc. to the authors’ own measurements using cold vapour atomic absorption spectrometry (CVAAS).

pH and electrical conductivity were measured using the multifunction meter (CX-505, Elmetron, Zabrze, Poland) with the electrode (range 2–12) (EPS-1, Elmetron, Zabrze, Poland) and the conductometer (range 10–1000 µS/cm) (EPS-2 ZE, Elmetron, Zabrze, Poland); anions were measured using the ion chromatograph with a conductivity detector (range 0.1–1000 mg/L) (Metrohm 930 Compact IC Flex, Metrohm, Herisau, Switzerland); total organic carbon was measured using spectrophotometer (range 3–65 mg/L) (DR 3800, Hach Lange, Düsseldorf, Germany); heavy metal was measured using ICP-OES (U5000AT+, CETAC, Omaha, NE, USA) with a pneumatic and ultrasonic nebuliser in the ranges: Cd 0.001–500 mg/L, As 0.003–100 mg/L, Zn 0.005–1000 mg/L, Cr 0.005–500 mg/L, Cu 0.005–1000 mg/L, Ni 0.004–500 mg/L, Pb 0.002–500 mg/L; and finally, mercury was determined using the atomic absorption spectrometer (AMA 254, Altec, Praha, Czech Republic) in the range of 0.0002–0.010 mg/L.

#### 2.1.2. Cladding Panel for Outdoor Applications

**Figure 2:**
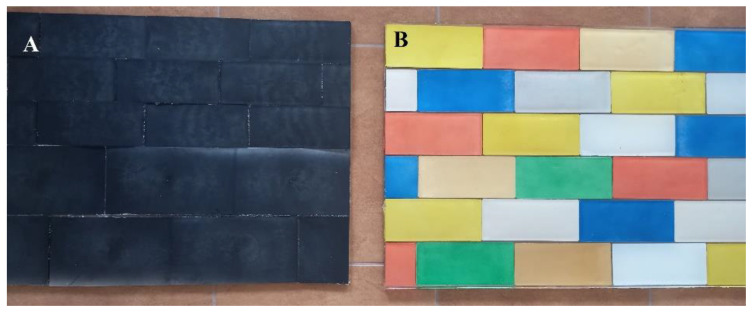
Cladding panel made from recycled polymer panels (**A**) and panel coated with a decorative layer as an example of application (**B**).

The first test demonstrator for the construction sector was designed as a cladding panel for outdoor applications made from recycled rPP/NP composite panels. Figure 2 presents a cladding panel used for further research (A) and a panel coated with a decorative (B). In order to evaluate the environmental impact of the rPP/NP, ion and heavy metal release, tests were performed. The tests were based on the methods described in CEN/TS 16637-2:2014 [28] and focused on the release of hazardous substances from construction products into surface water, groundwater and soil. The purpose of the tests was to determine the characteristics of the leaching process and to evaluate the release of environmentally harmful substances.

In the case of monolithic materials, i.e., the rPP/NP composite, the product specimen was placed in a leaching vessel with the surface to be exposed completely covered by the leachant. The test was carried out due to the use of the composite as a cladding panel for outdoor use. The leachant was poured into the vessel in a specified volume that was consistent with the surface area factor calculated from the size of the test specimen [28]. As in the case of the recycling material, the leachant was exchanged according to the frequency given in Table 2. The same physical properties were measured in the collected eluates as for the recyclates.

### 2.2. Testing for Emissions of Dangerous Substances into Indoor Air—Acoustic Barrier

Due to the intended use of acoustic barriers in rooms reserved for human use, the release of dangerous substances into the air is what reflects the impact of such barriers on the environment. VOC emissions from construction products cause indoor air pollution. VOC can be a major factor of indoor air pollution and affect the deterioration of the health and wellbeing of people in closed areas. Unified assessment of the VOC-related properties of indoor construction products reduces the risk of exposure of occupants to dangerous substances.

There are no normative requirements for acoustic barriers used indoors; thus, in order to assess their performance it is recommended to use the guidelines described in EN 1793-1:2017-05 [36] and EN 1794-1:2018-04 [37] concerning road traffic noise-reducing devices. These standards contain the acoustic and non-acoustic requirements for this type of product. 

VOC emission tests were conducted for the materials used to build the acoustic barriers, i.e., the rPE (HD:LD 50:50) + NP composite and the rPE (HD:LD 70:30) + NP composite for indoor applications.

The tests for the release of dangerous substances were performed in line with the following standards: PN-EN 16516:2017-11 [29], PN-EN ISO 16000-9:2009 [38], ISO 16000-6:2011 [39] and ISO 16000-3:2011 [40].

The tests refer to the emissions of volatile organic compounds and volatile aldehydes into indoor air. They involved placing the product in an emission chamber for 28 days under the following conditions:

- ventilated stainless-steel chamber
- temperature: (23 ± 1) °C
- relative humidity (50 ± 5)%
- air exchange rate 0.5 h^−1^
- saturation of the chamber with the product: 1 m^2^/m^3^

During the test, air samples were taken from the chamber twice: after 3 days and after 28 days from the start of the test. For the VOC test, approximately 5 L of air were collected, while for the volatile aldehyde tests, the volume of air collected was 60 L. The air samples were examined by chromatography:

- volatile organic compounds in the range C_6_–C_16_

VOCs were collected using glass tubes filled with Tenax TA adsorbent, which were desorbed using a thermal desorption unit (Shimadzu TD 20). VOCs’ separation and analysis were performed using gas chromatography with a mass detector GC/MS (Shimadzu GC/MS QP2020). VOCs were identified by comparing the chromatographic peaks’ retention times with those of the reference compounds and matching the resulting compounds’ spectra with those of the NIST 2011 database. Test range: 0.5–2000 µg/m^3^.

- volatile aldehydes in the range C_1_–C_4_

Air samples were taken to the collector cartridges with a solid absorbent, silica gel coated with 2,4-dinitrilophenyl hydrazine (2,4-DNPH), and then subjected to a laboratory test by high-performance liquid chromatography with a HPLC/UV (ultraviolet) detector (Thermo Fisher Scientific HPLC UltiMate 3000). Test range: 1–1000 µg/m^3^.

## 3. Results and Discussion

### 3.1. Testing for the Release of Dangerous Substances into the Environment

The results of leaching tests from recycled materials were compared with the criteria applicable to inert waste according to the Regulation of the Ministry of Economy of 16 July 2015 on the acceptance of waste at landfills [41]. A summary of the results together with the permissible values can be found in Table 3.

Tests on recycled materials have shown that, in most cases, the recyclates are environmentally friendly and can be reused. In the case of NP cellulose, the concentration limit was exceeded only for sulphate.

The leaching results of hazardous substances from recycled materials and the rPP/NP composite, presented in Table 4, were compared with the permissible values adopted for industrial wastewater discharged to water or the ground according to the Regulation of the Ministry of Maritime Affairs and Inland Navigation of 12 July 2019 [42].

The permissible values of TOC were exceeded only for NP cellulose. Taking into account the TOC value obtained for the rPP/NP composite, however, it can be concluded that NP cellulose can be used as a component of an environmentally safe composite. For the rest of the tested parameters, the permissible values were not exceeded.

### 3.2. Release Mechanisms of Hazardous Substances into the Environment

On the basis of test results for monolithic granular products and plate- or sheet-like products (DSLT test) of the rPP/NP composite, an analysis was carried out in order to identify the release mechanisms.

In those cases where the concentrations of substances leached at all steps of the test were low and close to the detection limit, it was not possible to determine the leaching process. The concentration of a substance is classified as low if:

(1) $$W1=\frac{c_{2-8}}{\mathrm{detection}\;\mathrm{limit}}<1.5$$

where: $c_{2-8\;}\;$ = $\frac{\sum_{8}^{i=2}c_{i}}{7}$. *c_i_*—concentration of the substance in eluate, in µg/L (mg/L). *c*_2–8_—average concentration of the substance in eluate 2 to 8, in µg/L (mg/L).

If the concentrations for an individual substance are too low, the determination of the other leaching mechanisms is omitted.

Surface leaching with low concentrations is a variant of dissolution, where there is relatively high leaching in the first (and second) eluate and low concentrations in the subsequent eluates. This case is considered when the following conditions are met:

(2) $$W2=\frac{c_{1}}{c_{3-7}}>1.8$$

(3) $$W3=\;\;\frac{c_{5-8}}{\mathrm{detection}\;\mathrm{limit}}<1.5$$

where: *c*_1_—concentration of the substance in the first eluate, in µg/L (mg/L). *c*_3–7_, *c*_5–8_—average concentration of the substance in eluates 3–7 and 5–8, in µg/L (mg/L). The results were presented in Table 5 and Table 6.

Based on the values of indicators W1, W2 and W3, it was not possible to determine the release mechanism for TOCs, chlorides, bromides and sulphates due to low concentrations that were close to the detection limit.

Based on the values of indicators W1, W2 and W3, it was not possible to determine the release mechanism for mercury, arsenic, chromium and nickel due to low concentrations that were below or close to the detection limit.

If the results do not correspond to the above cases, they are analysed in terms of diffusion mechanisms as the main release mechanisms. The level of diffusion depends on porosity and tortuosity. Diffusion occurs if:

(4) $$\sqrt{MSE}<0.40$$

*MSE*—mean square error of concentration in eluates 2–8, calculated using formulas from standard [28].

The mean square error is calculated in two cases: $\frac{c_{8}}{c_{7}}\;$ ≥ 0.9—no depletion and $\frac{c_{8}}{c_{7}}$ < 0.9—depletion.

Depletion occurs when there is a decrease in the concentration of the substance in the last fractions, and when the condition that the concentration in step 8 is lower than the concentration in step 7 is fulfilled.

If diffusion is determined as the primary release mechanism for an individual substance, it is necessary to check whether the secondary mechanism is surface leaching and/or depletion, according to: $\frac{c_{1}}{c_{3-4}}>1.8$. Then, diffusion determined as the primary process is preceded by surface leaching $\frac{c_{8}}{c_{7}}<0.9$. Diffusion is determined as the primary process but there is also depletion manifested by a decrease in concentration in the last fractions (7) and (8) respectively. If both of the above conditions are met, we are dealing with a mixed process involving diffusion as the primary process with surface leaching and depletion. A summary of release mechanisms in diffusion processes was presented in Table 7.

Based on the values of calculated indicators, it was concluded that the release of lead occurs due to the mixed process (diffusion as the basic process, and surface leaching and depletion). For the rest of the analysed substances, the value of the *MSE* (mean square error) was below 0.40, which allowed the exclusion of diffusion as a release mechanism.

If diffusion is not established as the main release mechanism, it is necessary to check whether dissolution may be the main release mechanism of the substance (Table 8). Dissolution [28] occurs if:

σ_pH_ < 0.25 and (5)

(6) $$\frac{\sigma_{c}}{c_{1-8}}<0.25$$

where:

(7) $$\sigma_{\mathrm{pH}}=\sqrt{\frac{\sum_{i=1}^{8}{\left(pH_{i}-pH_{1-8}\right)}^{2}}{8}}$$

(8) $${\mathrm{pH}}_{1–8}=\frac{\sum_{i=1}^{8}pH_{1-8}}{8}$$

(9) $$\sigma_{c}=\sqrt{\frac{\sum_{i=1}^{8}{\left(c_{i}-c_{1-8}\right)}^{2}}{8}}$$

where: *c_i_*—concentration of the substance in eluate, in µg/L (mg/L). *c*_2–8_—average concentration of the substance in eluates 2–8, in µg/L (mg/L).

Based on the values of the calculated indicators, it was concluded that the release of zinc occurs due to the dissolution process.

pH changes during the test can interfere with the release of the substance. Dissolution as the main process can only be identified through small differences in pH values in the eluates.

Another release mechanism is the surface leaching of a substance, which occurs when:

(10) $$\left|{\mathrm{pH}}_{1}-{\mathrm{pH}}_{2-8}\right|<0.5\;$$

(11) $$\mathrm{and}\;\frac{c_{1}}{c_{2-4}}>1.8$$

where: ${\mathrm{pH}}_{2-8}=\frac{\sum_{i=2}^{8}{\mathrm{pH}}_{i}}{7}$—average pH value of eluates 2 to 8. pH_i_—pH value of the eluate. $c_{2-4}=\frac{\sum_{i=2}^{4}c_{i}}{3}$—average concentration of the substance in eluates 2–4. *c_i_*—concentration of the substance in eluate i. If changes in pH are likely to interfere with substance release, surface leaching can only be identified if the pH difference between the first and subsequent eluates is limited. A summary of surface leaching processes was presented in Table 9.

Based on the values of calculated indicators, it was concluded that the release of copper and cadmium occurs due to the surface leaching process.

### 3.3. Emission of Hazardous Substances into Indoor Air

VOC emission tests were conducted for the polypropylene matrix composites reinforced with cellulose fibres (rPP/NP) and polyethylene-based composites reinforced with cellulose fibres (rPE (HD:LD 50:50) + NP, rPE (HD:LD 70:30) + NP) as materials used to build the acoustic barriers for indoor applications. The results were presented in Table 10.

The emission results obtained corresponded to national regulations on permissible concentrations of agents harmful to health in rooms intended for human occupancy, as found in the Ordinance of the Ministry of Health and Social Welfare of 12 March 1996 [43].

The ordinance distinguishes two types of rooms:

- category A—residential, intended for permanent residence of sick persons in healthcare facilities, and intended for permanent residence of children and young people in educational buildings, as well as rooms intended for the storage of food products.
- category B—public utility buildings other than those included in category A and auxiliary rooms in flats, intended for human occupancy.

Of the compounds included in the Ordinance [43], only two appeared in the results obtained from the emission tests of composites, i.e., 1-butanol and formaldehyde.

Annex 1 to [43] determines the permissible concentrations for some of the identified chemicals. They have been compared with the values obtained from tests of composite materials in Table 11. No permissible concentrations were determined for the other compounds listed. In summary, the composites tested did not exceed the permissible concentrations after 28 days of testing.

In addition, the test results can be cross-referenced to the requirements for harmful substances emitted by building products according to AgBB—Evaluation procedure for VOC emissions from building products [44]. They have been compared with the values obtained from tests of composite materials in Table 12.

A basis for these regulations is the EU directive on construction products, which requires that they do not pose any risk to building dwellers. The AgBB has assessed the observance of this clause of the relevant directive. The approach adopted by the AgBB describes minimum requirements for VOC emissions. Low-emission products can also be additionally labelled with voluntary marks such as Blue Angel, GUT, EMICODE etc. German authorities are working on the introduction of this assessment programme to the corresponding regulations for construction products across Europe.

## 4. Conclusions

1. There are no clear regulations describing environmental requirements for materials recycled from waste. The results of inorganic ion and heavy metal leaching tests were compared with the criteria for waste described in the Regulation of the Ministry of Economy on allowing waste to be placed at landfill sites [41].
2. Based on the results of inorganic ion and heavy metal leaching tests, recyclates in the form of rPE and rPP granulates can be considered environmentally friendly because they do not exceed the maximum permissible values. For NP cellulose, the maximum permissible sulphate concentrations were exceeded.
3. European legislation lacks regulations governing the acceptable environmental impact of composite cladding panels. The eluates from the test materials were treated as industrial wastewater entering the environment. By comparing the results with the guidelines of the Regulation of the Ministry of Maritime Economy and Inland Navigation [42], it was found that the leaching of the substances comprising the tested materials is not harmful to the environment, i.e., surface water, groundwater and soil.
4. For NP cellulose, the permissible TOC concentration was slightly exceeded (35.52 mg C/l) [42]. Nevertheless, when combining NP with a polymer material (rPP/NP composite), the TOC value was not exceeded, pointing to the possibility of using NP as composite reinforcement.
5. Following the analysis of mechanisms of release of dangerous substances from the rPP/NP composite, it was found that the release of lead is based on a mixed process, the main mechanism of which is diffusion-controlled; however, leaching and depletion also occur. The release of zinc is dissolution-controlled, while the release of copper and cadmium is based on the surface leaching process. It is impossible to determine the leaching mechanisms for the remaining tested parameters because the concentrations obtained were too low.
6. The tested composites meet the requirements of Polish regulations on the release of dangerous substances in accordance with [43] and may be used in category A and B rooms. In addition, permissible values were not exceeded when the results of the tests were compared with the European requirements described in the UE-LCI list, which is an appendix to the AgBB evaluation procedure [44].
7. The emission results for the rPP/NP composite point to its possible use as an indoor decorative panel.

## Figures and Tables

**Table 1:** List of secondary raw materials and composites subjected to environmental testing.

		Material	Designation
recyclates	granulates	Polyethylene regranulate	rPE
		Polypropylene regranulate	rPP
	cellulose	Cellulose obtained from newsprint	NP
	composites	Composite 1 with rPP matrix containing 30% vol. NP	rPP/NP
		Composite 2 with rPE matrix (LD:HD—50:50) containing 20% vol. NP	rPE (HD:LD 50:50) + NP
		Composite 3 with rPE matrix (LD:HD—30:70) containing 20% vol. NP	rPE (HD:LD 70:30) + NP

**Table 2:** Leachant exchange frequency according to [28].

Step	Step Duration	Duration from the Start of the Test
1	6 h	6 h
2	18 h	1 day (24 h)
3	1 day and 6 h (30 h)	2 days and 6 h (54 h)
4	1 day and 18 h (42 h)	4 days (96 h)
5	5 days (120 h)	9 days (216 h)
6	7 days (168 h)	16 days (384 h)
7	20 days (480 h)	36 days (864 h)
8	28 days (672 h)	64 days (1536 h)

**Table 3:** Comparison of test results with their permissible parameter values according to [41].

Indicator Name [Unit]	Permissible Value [41]	Average Value Obtained *		
		Recyclates		
		rPE	rPP	NP
TOC [mg C/l]	3000	7.93	4.35	35.52
F^−^ [mg F^−^/L]	1	0.09	0.08	0.08
Cl [mg Cl^−^/L]	80	0.60	1.60	3.43
SO_4_^2−^ [mg SO_4_^2−^/L]	100	0.17	0.46	472.06
Hg [mg Hg/L]	0.001	<4·10^−6^	<4·10^−6^	<4·10^−6^
Cd [mg Cd/L]	0.004	<4·10^−5^	<4·10^−5^	2·10^−4^
As [mg As/L]	0.05	<1·10^−3^	2·10^−3^	2·10^−3^
Zn [mg Zn/L]	0.4	5·10^−5^	6·10^−2^	2·10^−2^
Cr _total_ [mg Cr/L]	0.05	<1·10^−4^	<1·10^−4^	9·10^−4^
Cu [mg Cu/L]	0.2	4·10^−4^	1·10^−3^	1·10^−3^
Ni [mg Ni/L]	0.04	1·10^−6^	1·10^−6^	3·10^−3^
Pb [mg Pb/L]	0.05	<4·10^−5^	<4·10^−5^	8·10^−3^

* Weighted average, where weights are stage durations from Table 2.

**Table 4:** Comparison of test results with their permissible parameter values according to [42].

Indicator Name [Unit]	Permissible Value [42]	Average Value Obtained *			
		Recyclates			RPP/NP Composite
		rPE	rPP	NP	
Temperature [°C]	35	23.10	23.08	23.01	22.59
pH [no unit]	6.5–9	7.71	7.60	7.97	6.82
TOC [mg C/L]	30	7.93	4.35	35.52	0.99
F^−^ [mg F^−^/L]	25	0.09	0.08	0.08	0.02
Cl^−^ [mg Cl^−^/L]	1000	0.60	1.60	3.43	0.01
SO_4_^2−^ [mg SO_4_^2−^/L]	500	0.17	0.46	472.06	0.12
Σ Cl^−^ + SO_4_^2−^ [mg Cl^−^/L + SO_4_^2−^/L]	1500	0.76	2.06	475.49	0.13
Hg [mg Hg/L]	0.03	<4·10^−6^	<4·10^−6^	<4·10^−6^	<4·10^−6^
Cd [mg Cd/L]	0.2	<4·10^−5^	<4·10^−5^	2·10^−4^	6·10^−6^
As [mg As/L]	0.1	<1·10^−3^	2·10^−3^	2·10^−3^	<1·10^−3^
Zn [mg Zn/L]	2	5·10^−5^	6·10^−2^	2·10^−2^	1·10^−2^
_Total_ Cr [mg Cr/L]	0.5	<1·10^−4^	<1·10^−4^	9·10^−4^	<1·10^−4^
Cu [mg Cu/L]	0.5	4·10^−4^	1·10^−3^	1·10^−3^	9·10^−4^
Ni [mg Ni/L]	0.5	1·10^−6^	1·10^−6^	3·10^−3^	2·10^−6^
Pb [mg Pb/L]	0.5	<4·10^−5^	<4·10^−5^	8·10^−3^	2·10^−2^

* Weighted average, where weights are stage durations from Table 2.

**Table 5:** Dynamic surface leaching test (DSLT)—pH, conductivity, anion concentration in eluates.

Step	pH	Conductivity [µS/cm]	TOC	Fluorides	Chlorides	Bromides	Sulphates
			Concentration mg/L				
c_1_	6.30	4.14	2.06	0.01	0.13	<0.1	0.15
c_2_	6.99	4.38	<1.0	0.01	<0.1	<0.1	<0.1
c_3_	6.67	4.35	<1.0	0.02	<0.1	<0.1	<0.1
c_4_	7.12	3.65	<1.0	0.01	<0.1	0.11	<0.1
c_5_	6.25	4.86	1.56	0.01	<0.1	<0.1	<0.1
c_6_	6.28	5.14	<1.0	0.02	<0.1	0.10	<0.1
c_7_	7.18	14.05	1.96	0.04	<0.1	<0.1	0.32
c_8_	6.79	6.31	<1.0	0.01	<0.1	0.11	<0.1
DL *	–	–	1.00	0.01	0.10	0.10	0.10
Low total concentrations—release mechanism cannot be determined							
W1 < 1.5	–	–	0.86	1.70	0.00	3.20	0.45
W2 > 1.8	–	–	0.70	0.50	0.00	0.00	0.00
W3 < 1.5	–	–	3.52	8.00	0.00	21.00	3.20

* Detection limit; – not applicable.

**Table 6:** DSLT test—concentration of metals in eluates.

Step	Hg	Cd	As	Zn	Cr	Cu	Ni	Pb
	Concentration µg/L							
1	<0.004	0.17	<1	16.10	<0.1	1.44	0.49	3.07
2	<0.004	0.09	<1	11.70	<0.1	<0.2	<0.1	1.03
3	<0.004	0.10	<1	16.60	<0.1	<0.2	<0.1	1.74
4	<0.004	0.09	<1	15.80	<0.1	<0.2	<0.1	1.06
5	<0.004	0.06	<1	13.20	<0.1	0.32	<0.1	1.78
6	<0.004	0.04	<1	9.64	<0.1	0.42	<0.1	1.61
7	<0.004	0.06	<1	12.60	<0.1	1.28	<0.1	1.89
8	<0.004	0.06	<1	11.30	<0.1	0.87	<0.1	1.09
DL *	0.004	0.04	1.00	0.20	0.10	0.20	0.10	0.40
Low total concentrations—release mechanism cannot be determined								
W1	0.00	1.80	0.00	46.90	0.00	2.30	1.00	3.60
W2	–	–	–	0.67	–	0.61	2.80	1.90
W3	–	–	–	58.00	–	1.15	1.00	1.60

* Detection limit; – not applicable.

**Table 7:** Evaluation of the occurrence of release mechanisms in diffusion processes.

Substance	Indicators				
	$\sqrt{MSE}$	$\frac{c_{8}}{c_{7}}$	$\frac{c_{8}}{c_{7}}$	$\frac{c_{1}}{c_{3-4}}$	Diffusion
Fluorides	0.56	4	−	0.67	−
Cadmium	0.76	1	−	1.8	−
Copper	0.92	−	0.68	7.7	−
Lead	0.28	−	0.28	2.21	+
Zinc	0.45	0.9	−	0.99	−
Requirement	<0.40	≥0.9 no depletion	<0.9 depletion	>1.8	−

+: diffusion occurs; −: no diffusion.

**Table 8:** Evaluation of dissolution as a release mechanism.

Substance	Indicators		Dissolution
	σ_pH_	σ_c_/*c*_1–8_	
Fluorides	0.16	0.61	–
Cadmium		0.44	–
Copper		0.51	–
Zinc		0.18	+
Requirement	<0.25	<0.25	–

+: dissolution occurs; −: no dissolution.

**Table 9:** Evaluation of the occurrence of surface leaching processes.

Substance	Indicators		Dissolution
	pH_1_—pH_1–8_	*c*_1_/*c*_2–4_	
Fluorides	0.45	0.77	−
Cadmium		1.8	−/+
Copper		4.8	+
Requirement	<0.5	>1.8	−

+: dissolution occurs; −: no dissolution.

**Table 10:** Emissions of hazardous substances from composites for indoor applications.

Identified Chemical Compound	[CAS]	Concentration after 3/28 Days [µg/m^3^]		
		rPP/NP	rPE (HD:LD 50:50) + NP	rPE (HD:LD 70:30) + NP
Hexane	[110-54-3]	15/18	2/<1	80/<1
n-Octane	[111-65-9]	−	−	6/2
2,2,4-Trimethylhexane	[16747-26-5]	−	−	3/<1
2,2-Dimethylheptane	[1071-26-7]	4/<1	−	−
3,3,5-Trimethylheptane	[7154-80-5]	−	−	6/<1
2,2-Dimethyldecane	[17302-37-3]	−	−	8/<1
2,6,10-Trimethyldodecane	[3891-98-3]	−	−	4/<1
Dodecane	[112-40-3]	−	−	4/<1
Tetradecane	[629-59-4]	−	−	5/<1
Pentadecane	[629-62-9]	4/<1	−	−
Butan-1-ol	[71-36-3]	−	9/7	−
2-Ethyl-1-hexanol	[104-76-7]	8/<1	−	−
2-Propylheptan-1-ol	[10042-59-8]	−	9/9	−
Cyclodecanol	[1502-05-2]	7/<1	−	−
2-Methoxypropene	[116-11-0]	9/4	−	−
Nonanal	[124-19-6]	−	2/<1	5/<1
α-Pinene	[80-56-8]	−	−	7/3
3-Carene	[13466-78-9]	−	−	8/<1
1-(2-butoxy-1-methylethoxy)propan-2-ol	[29911-28-2]	−	−	2/<1
Number of unidentified chemical compounds		6/8	−	4/<1
Total Volatile Organic Compound (TVOC)		51/30	30/11	111/3
Formaldehyde	[50-00-0]	<1/<1	4/<1	<1/<1
Acetaldehyde	[75-07-0]	<1/<1	2/<1	<1/<1
Propionaldehyde	[123-38-6]	<1/<1	12/<1	<1/<1
Butyraldehyde	[123-72-8]	<1/<1	<1/<1	<1/<1

−: not detected.

**Table 11:** Permissible concentrations of chemical substances harmful to health in the air of category A/B rooms according to [43] with average concentrations of substances emitted from the tested composites.

Identified Chemical Compound	Average Concentrations of Substances in Chamber Air after 28 Days [µg/m^3^]			Permissible Value in μg/m^3^ in Category A/B Rooms
	rPP/NP	rPE (HD:LD 50:50) + NP	rPE (HD:LD 70:30) + NP	
Butan-1-ol	−	7	−	300/300
Formaldehyde	<1	<1	<1	50/100

−: not detected.

**Table 12:** Test results after 28 days and permissible concentrations related to European requirements found in the European Union (EU) LCI list, which constitutes an annex to the German Committee for Health-Related Evaluation of Building Products (AgBB) procedure [44].

Name of the Substance	[CAS]	Permissible Concentration [µg/m^3^]	Concentration after 28 Days [µg/m^3^]		
			rPP/NP	rPE (HD:LD 50:50) + NP	rPE (HD:LD 70:30) + NP
Hexane	[110-54-3]	4300	18	<1	<1
n-Octane	[111-65-9]	14,000	−	−	2
2,2-Dimethylheptane	[1071-26-7]	14,000	<1	−	−
2,2,4-Trimethylhexane	[16747-26-5]	14,000	−	−	<1
2,6,10-Trimethyldodecane	[3891-98-3]	6000	−	−	<1
2,2-Dimethyldecane	[17302-37-3]	6000	−	−	<1
3,3,5-Trimethylheptane	[7154-80-5]	14,000	−	−	<1
Dodecane	[112-40-3]	6000	−	−	<1
Tetradecane	[629-59-4]	6000	−	−	<1
Pentadecane	[629-62-9]	6000	<1	−	−
Butan-1-ol	[71-36-3]	3000	−	7	−
2-Ethyl-1-hexanol	[104-76-7]	300	<1	−	−
Nonanal	[124-19-6]	900	−	<1	<1
α-Pinene	[80-56-8]	2500	−	−	3
3-Carene	[13466-78-9]	1500	−	−	<1
1-(2-butoxy-1-methylethoxy)propan-2-ol	[29911-28-2]	250	−	−	<1
TVOC		1000	30	23	3
Formaldehyde	[50-00-0]	100	<1	<1	<1
Acetaldehyde	[75-07-0]	1200	<1	<1	<1
Propionaldehyde	[123-38-6]	650	<1	<1	<1
Butyraldehyde	[123-72-8]	650	<1	<1	<1

−: not detected.

## Data Availability

Not applicable.
